# Optimal Design of a Center Support Quadruple Mass Gyroscope (CSQMG) †

**DOI:** 10.3390/s16050613

**Published:** 2016-04-28

**Authors:** Tian Zhang, Bin Zhou, Peng Yin, Zhiyong Chen, Rong Zhang

**Affiliations:** Engineering Research Center for Navigation Technology, Department of Precision Instruments, Tsinghua University, Beijing 100084, China; zhangtian13@mails.tsinghua.edu.cn (T.Z.); yinp14@mails.tsinghua.edu.cn (P.Y.); chendelta@mail.tsinghua.edu.cn (Z.C.)

**Keywords:** rate MEMS gyroscope, symmetric structure, quadruple mass, center support, coupling beam, optimal design

## Abstract

This paper reports a more complete description of the design process of the Center Support Quadruple Mass Gyroscope (CSQMG), a gyro expected to provide breakthrough performance for flat structures. The operation of the CSQMG is based on four lumped masses in a circumferential symmetric distribution, oscillating in anti-phase motion, and providing differential signal extraction. With its 4-fold symmetrical axes pattern, the CSQMG achieves a similar operation mode to Hemispherical Resonant Gyroscopes (HRGs). Compared to the conventional flat design, four Y-shaped coupling beams are used in this new pattern in order to adjust mode distribution and enhance the synchronization mechanism of operation modes. For the purpose of obtaining the optimal design of the CSQMG, a kind of applicative optimization flow is developed with a comprehensive derivation of the operation mode coordination, the pseudo mode inhibition, and the lumped mass twisting motion elimination. The experimental characterization of the CSQMG was performed at room temperature, and the center operation frequency is 6.8 kHz after tuning. Experiments show an Allan variance stability 0.12°/h (@100 s) and a white noise level about 0.72°/h/√Hz, which means that the CSQMG possesses great potential to achieve navigation grade performance.

## 1. Introduction

In view of the fact microelectromechanical systems (MEMS) technology is rapidly developing, it has been possible to create gyroscopes with small size, low weight, low cost, and low power under the premise of high precision. Therefore, MEMS gyros are used not only in high precision applications such as aerospace and military, but also a wide range of consumer market uses including cellphones, automotive, and intelligent wear [1,2].

MEMS vibratory gyroscopes generally utilize Coriolis’ force to sense angular velocity. To operate the CSQMG under drive mode, when an angular velocity is introduced, the moving part of the gyroscope experiences a Coriolis force, and the gyro induces a sense mode oscillation. The input angular velocity can then be measured by detecting the sense mode vibration [3]. Most MEMS gyros are of the vibratory type, and high sensitivity dual-mass Tuning Fork Gyros (TFGs) play an important role. In the tuning fork design, linear acceleration/shock signals are rejected by its symmetric tuning fork architecture [4,5], however, the performance and environmental robustness are limited by the asymmetric sense mode [6,7].

Since the fully symmetric structures with higher performance were identified in recent years, research on micro 3-D symmetric structures has been emphasized. Micro-hemispheric structures have been demonstrated to achieve high Q and high precision potential, while many different manufacturing processes such as glass blowing, silicon etching, and integrated precision machining have been studied to create high precision 3-D hemispherical resonators [8,9,10,11]. As the exploration of new fabrication technology has made the micro HRG become a new research hotspot, the development potential of symmetrical structures has come to be highly regarded at the same time [12].

A new coupling design of MEMS tuning-fork structures, Quad Mass Gyro (QMG) sealed with getter, was proposed to approach gyro compassing and navigation grade performance [13]. The design of the coupling beam, which creates a high frequency separation between anti-phase and in-phase vibratory modes, and balanced operation mode eliminates energy dissipation through the substrate [14,15,16,17]. However, this design causes more folded beams, which introduce more error factors and consequently increase the design and fabrication difficulty [18].

**Figure 1:**
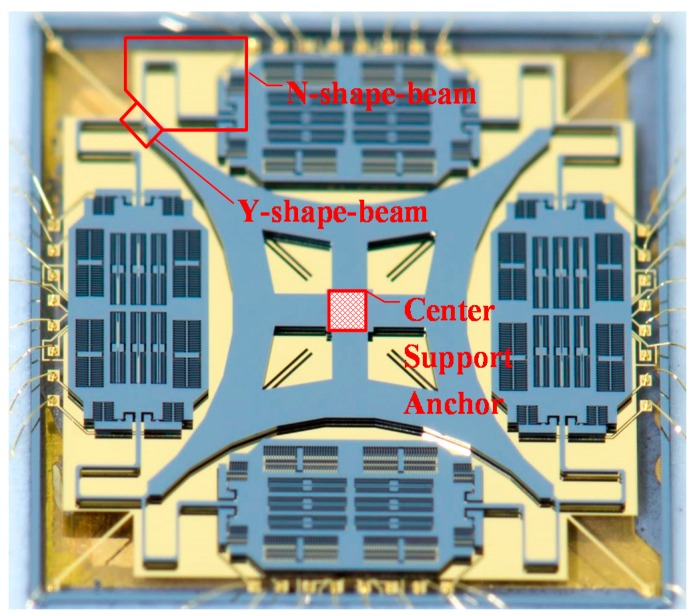
Optical photograph of a Center Support Quadruple Mass Gyroscope, with four lumped masses connected through the N-shape-beams and the Y-shape-beams, supported by a single center support anchor.

Considering the increasing maturity of the highly batch consistent Silicon-on-Glass/Silicon-on-Insulator (SOG/SOI) planar process, rational MEMS gyro structure pattern with symmetric form is a feasible development direction to achieve breakthrough performance [19]. Recently, a new structure adopting a simpler flexure pattern and a single center support anchor to support four proof masses was introduced [20], which integrates the advantages of TFG (large mass and high resolution) and HRG (fully symmetric and high Q) into a single flat structure (shown in Figure 1). Compared to conventional flat structures, the new design forms a fully symmetric configuration, and adjusts the mode frequency distribution through serpentine beams. Therefore, the fabrication process robustness is enhanced.

Besides, the Y-shaped coupling beam component is introduced to solve the problem of in-phase and anti-phase mode frequency confounding, and this design enhances the synchronization mechanism of operation modes. Because of its simple structure, without introducing complicated structures, the CSQMG achieves similar operation modes to HRGs (0° and 45° vibration modes) by its double tuning fork configuration. Thus the CSQMG can achieve the same environmental robustness as HRGs.

Along with the benefits of the new design, the parameters of a single beam affect the stiffness acting on more than one direction, which means more factors should be considered in the traditional structure optimization processes. At the same time, the lumped masses with two support points can be affected by torsion coupling stiffness. Motivated by the detailed analysis of the structure, a comprehensive derivation of the new design and the function of the serpentine beams is required to show the design rationality. An optimization flow considering multi-factor needs to be put forward in order to simplify the design.

In this paper, a more complete description of the CSQMG is presented. In Section 2, a description of the quadruple mass oscillator architecture with its operation mode is discussed. Section 3 presents a comprehensive derivation of the structure and the function of the serpentine beams, and this model is evaluated by Finite-Element Analysis (FEA). An optimization flow based on the model is concluded, and the comprehensive design parameters of the gyro are given in Section 4. Afterwards, the fabrication process and experimental characterization are presented in Section 5. Section 6 concludes this paper with a discussion and summary of the results.

## 2. Operation Mode

**Figure 2:**
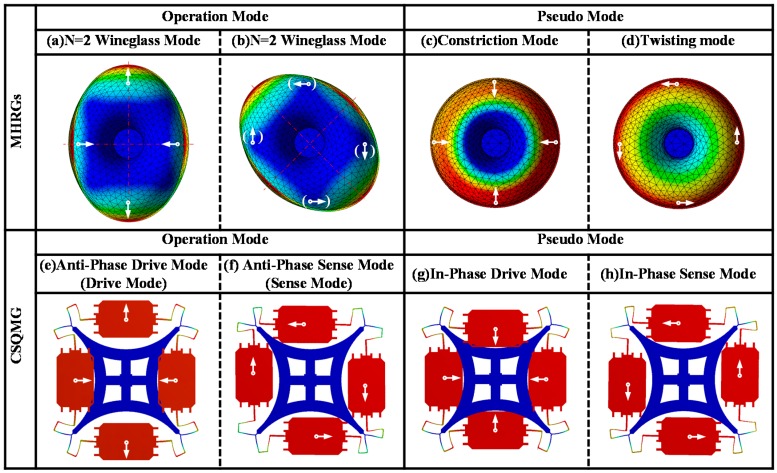
FEA shows the operation modes and pseudo modes of CSQMG, comparing to the resonant mode of the HRGs. In the operating modes of CSQMG, also known as anti-phase modes, the four lumped masses vibrate along the radial of the symmetry center in the drive mode, and vibrate along the tangential orientation under the sense mode. Moreover, the two adjacent lumped masses oscillate in anti-phase motion. Corresponding to the operation modes, the two in-phase modes are regarded as the pseudo modes.

The inspiration of CSQMG comes from HRGs, especially the *n* = 2 wine glass mode, as depicted in Figure 2. In order to transform the HRGs into a planar structure, the 0°/90° resonance mode and the −45°/45° resonance mode of the *n* = 2 wineglass mode are regarded as drive mode and sense mode, respectively. Then the hemisphere structure is simplified to planar structure by the following two steps. Firstly, as for drive mode, the distributed mass is concentrated at the harmonic displacement peak of the hemisphere resonator, forming four lumped masses. The four placement points of the resonator are supported through the bracing structure forming four fixed points (illustrated in Figure 2a,e). Secondly, as for sense mode, considering the tangential motion component of hemisphere resonator mass elements, the resonant elements are turned into four lumped masses alternating towards and away from the central point (illustrated in Figure 2b,f).

Though the latter transformation can’t match the sense mode resonance perfectly, the hemispherical resonator is thus creatively transformed into an in-plane dual tuning fork resonator. It is obvious that, when the quadruple mass resonator works as a gyro, the four lumped masses vibrate along the radius of the symmetry center in the drive mode and vibrate along the tangential orientation under the sense mode. Moreover, in the polar coordinates reference frame, the two adjacent lumped masses oscillate in anti-phase motion, which is the same as tuning fork anti-phase mode. Compared to conventional dual-mass TFGs, the major advantage of this symmetric CSQMG is the 4-fold symmetrical axes pattern. With in-plane dual tuning fork resonator and differential signal extraction, the common mode linear and centrifugal interferences such as acceleration and shock are rejected.

In drive mode, the lumped mass is restrained by the bilateral symmetrical structure and only radial motion is permitted. However, a coupling component along the lumped mass twisting motion potentially exists during the sense mode resonance, and it should be eliminated during the design optimization process.

Accompanied by the transformation, in-phase modes are also inevitable in CSQMG. Instead of vertical mode and tilting mode acting as major pseudo modes of HRGs, “constriction mode” and “twisting mode” become the major pseudo modes of the new structure. In constriction mode, the resonance alternates between contraction and expansion (depicted in Figure 2c,g), while in twisting mode, the resonance alternates between clockwise and counterclockwise (depicted in Figure 2d,h). Although the gyro can also operate in in-phase modes, the common mode signal cannot be eliminated.

The application of the N-shaped beam and the Y-shaped coupling beam enables a vibration of two in-plane quadrature directions with a single resonant beam [15]. Collectively, the four lumped masses, four resonant beams and four fixed points form a circular resonator, and the optimized distribution of each mode’s natural frequency is obtained by changing the design parameters.

**Figure 3:**
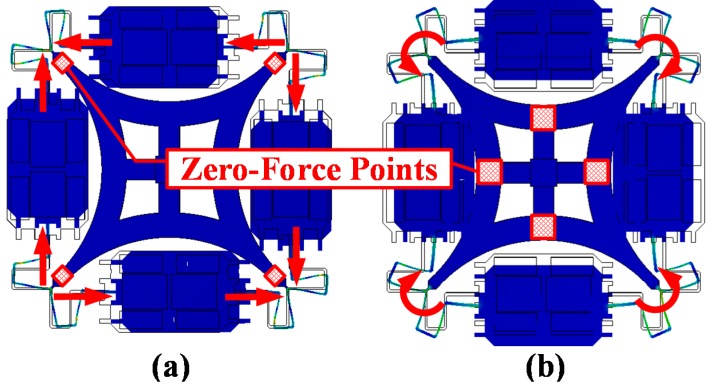
FEA shows the zero-force points of the operation modes. (**a**) Symmetric generalized force is exerted on the four fixed point during sense mode resonance, and the four placement points of the circular resonator are location points; (**b**) A mat-shaped bracing structure is designed to eliminate the placement point torque at the four center points of each curved beam, and these points become zero-force points in the drive mode.

The bracing structure is transformed into a single anchor support form in order to achieve low anchor loss [21], like HRGs [9]. As the four placement points of the circle resonator simplify the support form, a symmetric generalized force is exerted on the four fixed points during the sense mode resonance. In the ideal situation, the four points are regarded as zero-force points in the sense mode (Figure 3a). While in drive mode, because of the anti-symmetric generalized force, such an equivalence is no longer applicable, so a mat-shaped bracing structure is designed to eliminate the placement point torque at the four center points of each curved beam. These points become zero-force points in the drive mode (Figure 3b). Therefore, the single center supporting anchor is regarded as a zero-force point in both operation modes.

## 3. Theoretical Model

As mentioned in Section 2, on account of the N-shaped beam and the Y-shaped coupling beam design, the four lumped masses oscillate synchronously as a single circle resonator in comparison to conventional TFGs. Unlike the usual folded beam that determines stiffness of only one orientation, this design enables a vibration of two in-plane quadrature directions with a single resonant beam. This makes it difficult to establish a second-order system equation for the single resonant mass. As there are no referential design guidelines, the relationship between the geometry and the frequency distribution of the modes needs to be confirmed.

### 3.1. Derivation of the Flexibility Matrix

Taking account of the 4-fold symmetrical axes pattern, we simplify the analysis as a symmetric model with the following assumptions [18]:

- The lumped mass is regarded as a rigid body of length 2q, and all of the generalized forces are concentrated in the barycenter of the mass.
- The mass of the beams is neglected and the placement points are treated as fixed during oscillation.
- Following the hypotheses of linear strain and small deformation, and the effect of tension and compression stresses is neglected in the analysis process.
- As the Y-shaped coupling beam and barycenter of the lumped mass are symmetric axes of the structure, we presume an identical symmetric or anti-symmetric orthogonal generalized force is applied on both sides.

**Figure 4:**
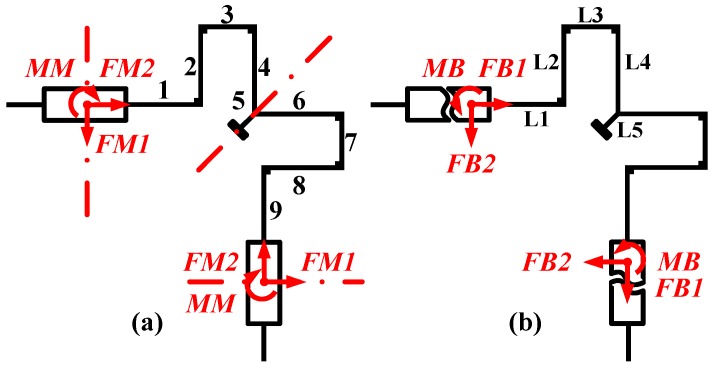
Simplified mechanics model of a quarter of the circle resonator. (**a**) Simulating the operation mode under external forces, and exerting external generalized unit forces FM1, FM2, MM. Numbering the microbeams from No. 1 to No. 9. Identifying the axes of symmetric position with a dotted line; (**b**) Naming the accompanied internal generalized unit forces FB1, FB2, and MB, when the circle resonator is under external forces.

**Figure 5:**
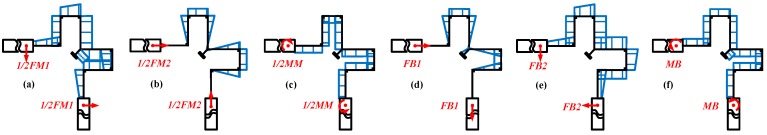
The bending moment diagram of the circle resonator simplified mechanics model after removing the constraints at the lumped masses symmetric axes. (**a**) Simulating external forces under the anti-phase drive mode; (**b**) Simulating external forces under the anti-phase sense mode; (**c**) Simulating external forces under lumped mass twisting motion coupling with anti-phase sense mode; (**d**) Assuming internal forces along the tangential orientation; (**e**) Assuming internal forces along the radial of the symmetric center; (**f**) Assuming internal forces along the lumped mass twisting motion.

The simplified model is still a statically indeterminate system. We solve the system with the statics flexibility method [22,23], remove the constraint at the lumped masses symmetric axes, exert external generalized unit forces FM1, FM2, MM and assume internal generalized unit forces FB1, FB2, MB, as shown in Figure 4. Then the canonical equation of deformation (1) based on the virtual displacement principle is established, and the torque expressions x_*ak*_~x_*fk*_ (*k* ∈ [1, 9] ∩ *k* ∈ *N*) are listed in Table 1 based on Figure 5.

(1) $$\sum_{k=1}^{9}\frac{1}{E_{k}I_{k}}\int_{0}^{lk}\left(\begin{array}{ccc} x_{dk}^{2} & x_{dk}x_{ek} & x_{dk}x_{fk}\\ x_{dk}x_{ek} & x_{ek}^{2} & x_{ek}x_{fk}\\ x_{dk}x_{fk} & x_{ek}x_{fk} & x_{ek}^{2} \end{array}\right)dx_{k}\cdot \left(\begin{array}{c} X_{p1}\\ X_{p2}\\ X_{p3} \end{array}\right)+\sum_{k=1}^{9}\frac{1}{E_{k}I_{k}}\int_{0}^{lk}\left(\begin{array}{c} x_{pk}x_{dk}\\ x_{pk}x_{ek}\\ x_{pk}x_{fk} \end{array}\right)dx_{k}=0$$

In the canonical Equation (1), *X*_*p*1_, *X*_*p*2_, *X*_*p*3_ (*p* = a, b, c) represent the internal forces under the action of external force FM1 (*p* = a), FM2 (*p* = b), MM (*p* = c), respectively.

Internal generalized unit forces are obtained by solving the canonical equation. Based on Castigliano’s second theorem, the flexibility matrix derivation of operation modes (anti-phase modes) is formulated, as illustrated in Equation (2):

(2) $$\left(\begin{array}{c} x\\ y\\ \theta \end{array}\right)=\left(\begin{array}{ccc} S_{11} & 0 & 0\\ 0 & S_{22} & S_{23}\\ 0 & S_{32} & S_{33} \end{array}\right)\left(\begin{array}{c} F_{x}\\ F_{y}\\ M \end{array}\right)\begin{array}{cc} & \begin{array}{l} S_{11}=\sum_{k=1}^{9}\frac{1}{E_{k}I_{k}}\overset{lk}{\underset{0}{\int }}{\left(x_{ak}+X_{a1}x_{dk}+X_{a3}x_{fk}\right)}^{2}dx_{k}\\ S_{22}=\sum_{k=1}^{9}\frac{1}{E_{k}I_{k}}\overset{lk}{\underset{0}{\int }}{\left(x_{bk}+X_{b2}x_{ek}\right)}^{2}dx_{k}\\ S_{33}=\sum_{k=1}^{9}\frac{1}{E_{k}I_{k}}\overset{lk}{\underset{0}{\int }}{\left(x_{ck}+X_{c2}x_{ek}\right)}^{2}dx_{k}\\ S_{23}=S_{32}=\sum_{k=1}^{9}\frac{1}{E_{k}I_{k}}\overset{lk}{\underset{0}{\int }}\left(x_{ck}+X_{c2}x_{ek}\right)\left(x_{bk}+X_{b2}x_{ek}\right)dx_{k} \end{array} \end{array}$$

*S_11_* represents the flexibility of anti-phase drive orientation, *S_22_* represents the flexibility of anti-phase sense orientation, and *S_33_* represents the flexibility of lumped mass torsion orientation. It’s noteworthy that *S_23_* is non-vanishing which means a coupling component along the lumped mass twisting motion exists in the sense mode. This is an undesirable characteristic in this structure as well, which should be considered in the subsequent optimization flow.

For in-phase modes, the symmetry of internal forces and external forces interchange. By changing the external force orientations with the internal force orientations, listing torque expressions, and using the same calculation steps, the flexibility matrix derivation of pseudo modes is also built.

### 3.2. FEM Validation of Theoretical Model

**Figure 6:**
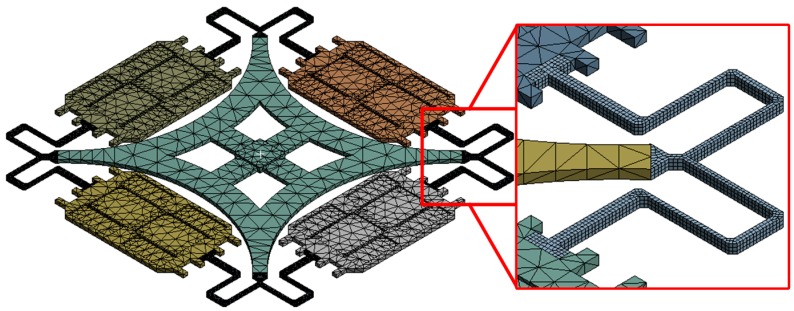
The finite element model of CSQMG with 35 μm chamfer combining two different mesh size. The comb fingers are replaced by evenly distributed mass. Swept meshing with element size less than 0.02 mm is used on the beams.

For the purpose of verifying the accuracy of the theoretical model, the geometric parameters obtained by optimization method are ported into FEM software ANSYS, as depicted in Figure 6. To reduce the amount of computation, the model is meshed with two kinds of grid division. Swept meshing with element size less than 0.02 mm is used on the beams, and default values with element size less than 0.2 mm is adopted for the rest. To reduce unnecessary division, the comb fingers are replaced by an evenly distributed mass as well. Static load is applied in accordance with the resonance form of the CSQMG, by means of fixing the single anchor and exerting a unit force on the lumped mass.

The average stiffness error of the four modes is less than 4%, proving the accuracy of the modeling process (Table 2). The main errors of the model come from the connection points of each beams which do not fit the assumptions during the simulation process.

## 4. Design Optimization Process

Optimal natural frequency allocation of the CSQMG is the primal goal of the design optimization. In consideration of the length and width of the serpentine beams containing 18 variables and the varying degree of the parameter impacts, the analysis of the natural frequency configuration is divided into N-shaped beam and Y-shaped beam, respectively. To alleviate the optimization complexity, the control variables method is used to analyze the two components. Then a targeted independent optimization step to eliminate the coupling component between the sense mode motion and the lumped mass twisting motion is discussed. After the overall structure parameters are confirmed, an optimized design of the comb fingers based on reducing the mechanical noise is performed.

### 4.1. Optimal Design of the N-Shaped Beam

The model derived in Section 3.1 is used to the analysis of the N-shaped beams. Even taking no account of the effect of the Y-shaped coupling beam, the relationship between the static characteristics and the parameters of the beams are still complicated. The parameters contain a total of 16 variables, namely the width and the length of Beam No. 1 to No. 9 except Beam No. 5 (the Y-shaped coupling beam). Therefore, the model needs to be simplified for convenience of analysis. Further presuppositions are as follows:

- The parameters of serpentine beams on both sides of the symmetric axes are considered equal.
- The length of the beams is fixed for subsequent analysis later, as the stiffness is proportional to the length and the third power of the width, and the physical dimension is limited.
- The width of Beam No. 2 and Beam No. 4 are regarded identical, since the effect of Beam No. 2 and Beam No. 4 are almost the same.

**Figure 7:**
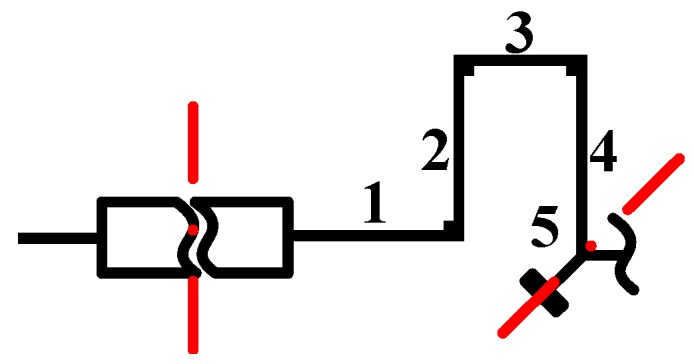
The further simplified model for optimal N-shaped beam design. We presuppose the parameters of the N-shaped beams on both sides of the symmetric axes are exactly the same.

**Figure 8:**
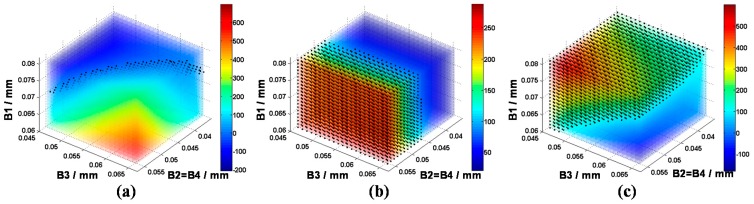
The relationship of geometric parameters B1,B2,B3,B4 and stiffness differences. (**a**) The stiffness difference between the two operation modes; (**b**) The stiffness difference between the in-phase drive mode and the anti-phase drive mode; (**c**) The stiffness difference between the in-phase sense mode and the anti-phase drive mode.

As shown in Figure 7, the simplifying assumptions above reduce the number of variables of the N-shaped beams from sixteen to three, *i.e.*, the width of Beam No. 1 (B1), the width of Beam No. 2 and No. 4 (B2 = B4), and the width of Beam No. 3 (B3). In order to show the variation trend between the N-shaped beams and the frequency distribution of the modes, a Cartesian coordinate system is established using the three variables, and the differences of the stiffness of every two different modes are shown using a color code. The Cartesian coordinate system forms three four-dimensional images, as shown in Figure 8.

Figure 8a shows the stiffness difference between the two operation modes. The red area means the stiffness of drive mode is larger than the sense mode, while the blue area indicates the opposite tendency. The closer to the red region, the larger the difference. As the CSQMG operates under the rate mode, the natural frequency of the drive mode should be set equal to or slightly higher than that of the sense mode. As shown in the figure, with the rise of B2, B3, B4 and the drop of B1, the stiffness difference between the two modes grows. The data points with stiffness differences greater than 0 but less than 50 N/m are marked with black spots as the Optimal Values Area 1 in the coordinate system. As the using of numerical methods limits the number of sample points, the range of black spots forms an approximate three-dimensional surface.

Figure 8b demonstrates the stiffness difference between the in-phase drive mode and the anti-phase drive mode. The red area means the stiffness of the in-phase drive mode is larger than the anti-phase drive mode. Shown from the figure, the stiffness difference is significantly affected by B2 and B4, but only slightly affected by B1 and B3. The difference becomes larger with the increase of B2 and B4. In order to get a gyro with better performance and reduce the impact of pseudo mode, the natural frequency of pseudo modes should be kept away from the operation mode. The data points with stiffness difference values greater than 100 N/m are marked with black spots forming Optimal Values Area 2.

Similar to Figure 8b, Figure 8c shows the stiffness difference between the in-phase sense mode and the anti-phase drive mode. The red area means the stiffness of the anti-phase drive mode is larger than the in-phase sense mode. The variation of B2 and B4 have less influence on stiffness changes, and with the increase of B1 and the decrease of B3 the difference become significant. The data points with stiffness difference values greater than 100 N/m are marked with black spots as the Optimal Values Area 3.

In summary, the variation of the parameter B1, B2, B3, and B4 changes the natural frequency distribution of each mode, and the variable ranges of the parameters are limited by the intersection of the three Optimal Values Area, especially the Optimal Values Area 1. In order to expand the scope, and increase the frequency difference between the operation modes and the pseudo modes, the Y-shaped coupling beam should be further analyzed.

### 4.2. Optimal Design of the Y-Shaped Coupling Beam

For the purpose of distinguishing each vibratory mode resonance frequency, a new type of Y-shaped coupling beam is put forward with the following advantages: (1) low relative machining error based on a simple structure; (2) in-phase modes separated from anti-phase modes with a single structure; (3) less energy is transforred to the substrate by locating it at the placement point.

**Figure 9:**
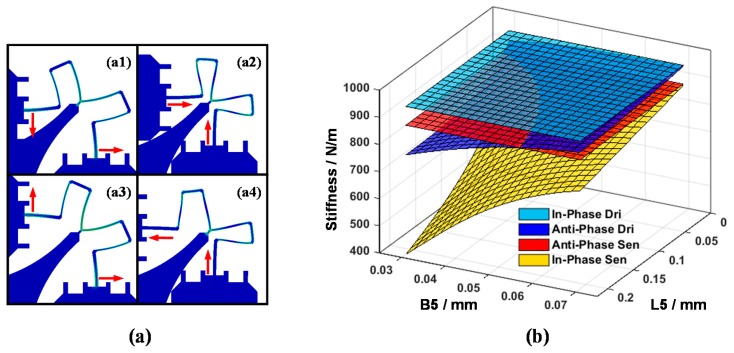
(**a**) The colored elastic strain patterns of Y-shaped coulping beam under anti-phase drive mode (**a1**); anti-phase sense mode (**a2**); in-phase drive mode (**a3**); and in-phase sense mode (**a4**); (**b**) The relationship of the Y-shaped beam geometric parameters and four main mode stiffness. Resonant stiffnesses between in-phase modes and anti-phase modes have been distinguished effectively by increasing the Y-shaped beam length and decreasing its width.

Connecting the support frame and the circle resonator at the placement points which act as symmetric axes, the Y-shape beam bears generalized forces from both sides. Considering the 4-fold symmetric axes pattern, an anti-symmetric generalized force is applied on both sides under anti-phase drive mode and in-phase sense mode, while a symmetric generalized force is applied in in-phase drive mode and anti-phase sense mode, as shown in Figure 9a. Intuitively, the design of the Y-shaped coupling beam changes the resonant stiffness of four planar modes.

In order to illustrate the influence of the Y-shaped beam more clearly, Figure 9b is presented. It is observed that by changing the Y-shaped beam parameters which include length increase and width decrease, the frequencies of the anti-phase drive mode and in-phase sense mode are shifted below the anti-phase sense mode. Similarly, with the parametric variation of beams, the stiffness of operation modes matches together, forming a range of optimal values. In addition, a small length value and width value are needed for low elastic damping loss purpose, while shifting above out-of-plane pseudo mode stiffness requires a large value of the parameters. Taking all these conditions into consideration, the optimal value range can be tightened to a narrow range.

### 4.3. Elimination of the Twisting Motion Coupling

As mentioned in Section 2, the *S_23_* of the flexibility matrix is non-vanishing, which means a coupling component exists between the lumped mass twisting motion and the sense mode motion. This phenomenon indirectly affects the coupling oscillation of drive mode and sense mode during the lumped mass planar motion, which deteriorates the performance of the CSQMG.

**Figure 10:**
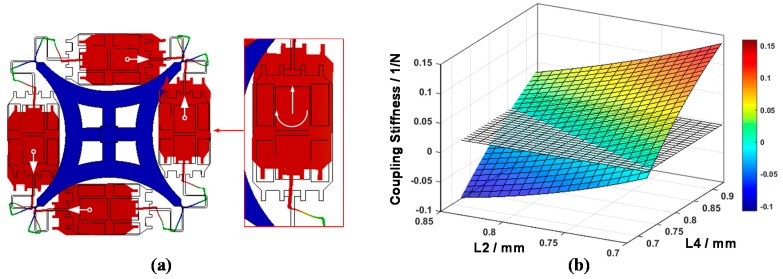
(**a**) Coupling component along the lumped mass twisting motion exists in the sense mode, which causes the coupling of the drive mode oscillation and sense mode oscillation; (**b**) The relationship of CSQMG’s geometric parameter L2, L4 and stiffness of twisting motion coupling. The twisting motion coupling can be eliminated by a reasonable allocation.

An optimization method should be applied to eliminate the coupling. Observing the simplified model, Beam No. 2 and No. 4 are perpendicular to the direction of sense mode vibration, and a forty-five-degree angle is formed between Beam No. 5 and the sense mode vibration. As the width of the beams and the parameters of the Beam No. 5 have been optimized in the above section, an optimization result should be provided via an analysis of the relationship between L2, L4 and the twisting motion coupling stiffness (Figure 10b). With the increasing of L2 and decreasing of L4, the coupling stiffness decreases and intersects the zero plane forming a straight line. This line is the optimal value range of L2 and L4 when the rest of the parameters are determined. Moreover, by means of changing the parameter of L2 and L4 and keeping the summation of these two parameters as a constant, a reasonable allocation is completed without disturbing the frequency distribution sharply.

### 4.4. FEM Validation of the Optimization Flow

Because chamfers are used at the connection points of each beams, the stress concentrations are reduced and fabrication process consistency is ensured. There is a slight discrepancy between the FEM analysis and the practical design. This discrepancy makes the optimization model unsuitable for the quantitative optimization task. However, the model is still effective in parameterized modeling as a qualitative optimization guide. Optimized analysis of the structure design proceeds through two steps: firstly, iterative calculation and analysis by changing the design of the N-shaped beam and Y-shaped beam are adopted, and these two conditions are taken into account at the same time: (a) the frequency of the drive mode should be slightly higher than the sense mode but no more than 20 Hz (considering about the range of electrostatic tuning) to tolerate the variation in the fabrication process; (b) the frequency of pseudo modes should be separated from the operation mode to more than 150 Hz to reduce the interference of pseudo modes. Secondly, L2 and L4 are changed and the summation of these two parameters is kept constant to find the minimum absolute value of the coupling component between the sense mode motion and the lumped mass twisting motion.

All the parameters of the actual CSQMG structure optimization are shown in Table 3, using the above two steps. By optimizing the width of the beams, the drive mode frequency is 2 Hz higher than the sense mode, the pseudo mode frequency is distinguished from the operation mode to 150 Hz and 434 Hz, respectively. Through optimizing the length of L2 and L4, the rotational coupling of sense mode is reduced from 0.022 N^−1^ to 4.2 × 10^−4^ N^−1^ while the frequency distribution is only slightly changed.

### 4.5. Optimal Design of Comb Fingers

For the conventional line vibration MEMS gyroscope, the main noise sources are as follows: the mechanical-thermal noise, the preamplifier noise, the digital quantization noise, and the ADC conversion noise. Compared to the mechanical-thermal noise, the digital quantization noise and the ADC conversion noise are a small amount in the CSQMG [24]. The mechanical-thermal noise of the conventional line vibration MEMS gyroscope is:

(3) $$\Omega_{th}=\frac{x_{noise}}{x_{\sec}(1{}^{\circ}/s)}=\frac{90}{\pi A_{0}\omega_{n1}}\sqrt{\frac{4\omega_{n2}k_{B}T}{mQ_{2}}}$$

where A_0_ is the amplitude of the drive mode, ω_*n*1_ is the natural frequency of the drive mode, ω_*n*2_ is the natural frequency of the sense mode, the k_*B*_ is the Boltzmann constant, T is the Kelvin temperature, m is the quality of lumped masses, and *Q*_2_ is the quality factor of the sense mode.

In order to achieve large displacements, which are necessary for low noise operation, 4 × 100 pairs of slide film comb fingers with a 40 μm long stroke are set in the driving direction [25]. Multi-comb fingers result in a maximum driving force of about 16 μN on each lumped mass, when the gyro operates at 10 V. 4 × 52 pairs of squeeze film comb fingers with differential capacitor type leading the sense direction possess over 3.3 pF (each lumped mass) total capacitance and 56 N/m stiffness tuning. The force balance electrodes with 4 × 14 pairs comb fingers guarantee 4 μN balance force at least. Chip size with footprint is 7700 μm × 7700 μm and the design parameters of the CSQMG are listed in Table 4. Substituting the parameters of the CSQMG, such as m, ω_*n*1_, ω_*n*2_, *Q*_2_, A_0_, into Equation (3), and the mechanical-thermal noise Ω_*th*_ is calculated to be 0.186°/h/√Hz.

## 5. Fabrication and Characterization

### 5.1. Fabrication Process

**Figure 11:**
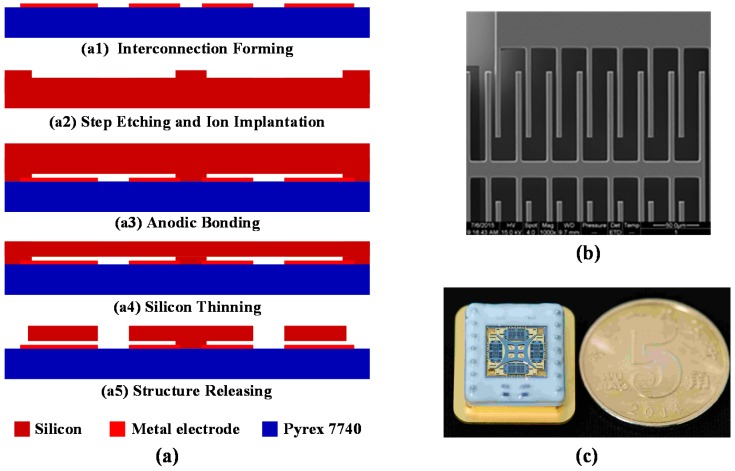
(**a**) Fabrication process of the CSQMG: (**a1**) Interconnection forming; (**a2**) Step etching and ion implantation; (**a3**) Anodic bonding; (**a4**) Silicon thining; (**a5**) Structure releasing; (**b**) SEM of CSQMG force balance electrodes. The gaps between the adjacent comb fingers are 4 μm and 16 μm; (**c**) Photograph of the CSQMG die in a metal case ready for experimental characterization.

A MEMS gyro sensitive structure fabrication process based on SOG and inductively coupled plasma deep reactive ion etching (ICP DRIE) with an aspect ratio of about 20 developed by Peking University is shown in Figure 11a [26,27,28]. The starting wafers are 4 inch highly doped silicon wafer produced by OKMETIC (Vantaa, Finland) and a Pyrex 7740 glass wafer. On the Pyrex wafer, a 200 nm thick Ti/Pt/Au layer is patterned by a liftoff process (Figure 11(a1)). On the silicon wafer steps of 20 μm height are etched by DRIE to define the anchor areas and the wafer is doped by phosphorus ion implantation to obtain good Ohmic contacts at the anchor areas (Figure 11(a2)). Afterwards the two wafer are bonded together (Figure 11(a3)). The silicon substrate is then thinned to about 100 μm using KOH (Figure 11(a4)) and released by a second DRIE step (Figure 11(a5)). Finally, the wafers are diced and wire bonded. Figure 11b,c show a SEM image and photograph of the fabricated gyroscope, respectively.

### 5.2. Frequency Response Curve Analysis

**Figure 12:**
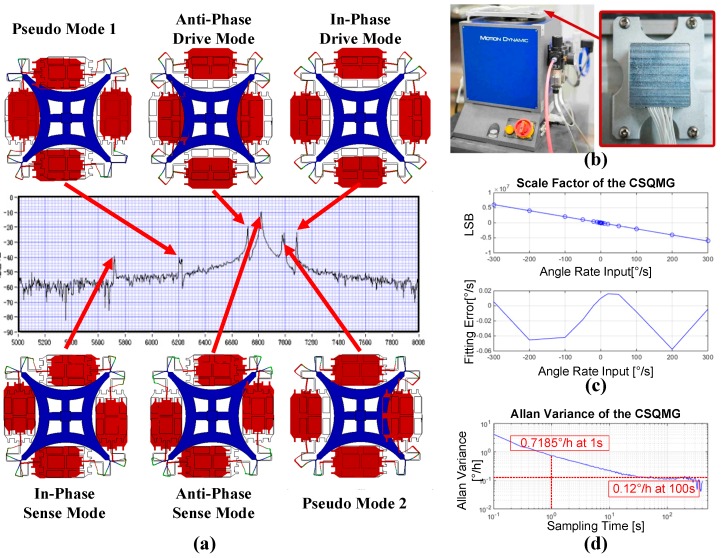
(**a**) Frequency response curve of the CSQMG. Sine excitations are applied on the drive combs and the signal read on sense combs, then all planar modes are reflected on the curve by vibration coupling; (**b**) Photograph of the Motion Dynamics 3V-AB 1-Axis Rate-Table (left) and fully packaged CSQMG (right); (**c**) Measured scale factor of the CSQMG. Above is the fitting result, below is the residual error. The scale factor is 20209.2LSB/(°/s) under ±300°/s measurement range, and nonlinearity is 96 ppm; (**d**) Measured Allan variance stability of the CSQMG. The white noise level is about 0.7185°/h/√Hz. The Allan variance stability is 0.12°/h (100 s) at room temperature.

Measurement of the gyro response curve is performed by applying sine excitations on drive combs and reading the signal on the sense combs under a pressure of 30 Pa [3]. Observing from the curve shown in Figure 12a, a total of six resonance peaks appear at 5.7, 6.2, 6.7, 6.8, 7.0 and 7.1 kHz approximately. Considering the influence of processing errors, each resonance peak matches the frequency of in-phase sense mode, pseudo mode 1, anti-phase drive mode (drive mode), anti-phase sense mode (sense mode), pseudo mode 2, and in-phase drive mode, respectively. Comparing to the FEM simulation results, the maximum deviation of the modal frequency is approximately 13.1%, and the deviation of the operation modes are 8.7% and 7.5%. The reductions of the modal frequency are attributed to the overetching during the fabrication process, and a much larger range of compensation of the beams width should be adopted in any subsequent improvement based on the theoretical model analysis. At the same time, due to the frequency response curve analysis, the theoretical model cannot reflect all of the CSQMG’s planar vibration information. Therefore, a mass-spring-damper model with multiple degrees of freedom should be put forward to simplify the CSQMG’s symmetric stiffness analyses, and a system parameter identification basing on experimental data needs to be studied in the future work.

### 5.3. Experimental Characterization

The CSQMG is packaged in the metal case, with a vacuum level of 30 Pa [20]. The quality factors of the drive mode and sense mode are measured to be about 8500 and 500, respectively. The key parameters of the CSQMG have been tested by a rate table from ACUITAS GmbH (Altendorf, Switzerland) as shown in Figure 12b. The MEMS and ASIC are assembled in an aluminum case, which is shown in Figure 12b. The gyro outputs are digitally transferred by RS422 protocol and collected by PC.

By using the rate table, the scale factor characterization can be evaluated [29]. This CSQMG’s measurement range is ±300°/s, and the scale factor nonlinearity is 96 ppm (Figure 12c). A 2 h static test was performed at room temperature and the results show that the white noise level is about 0.7185°/h/√Hz and the Allan variance stability is 0.12°/h (@100 s), (Figure 12d). Compared to the conventional TFGs, the CSQMG achieves a great performance improvement on account of its innovative structural design and its optimal design flow. According to the design parameters of the structure, shown in Table 5, the designed value of the mechanical noise level is about four times smaller than the measurement result, which means the main noise of the CSQMG is caused by the circuits, and the measured white noise level could be reduced by improving the performance of the electronics.

## 6. Conclusions

This paper has proposed and demonstrated a new double tuning fork gyro with a single support and 4-fold symmetric axes. The test results, especially the white noise level, shows the CSQMG has great potential to achieve navigation grade performance. As proved by both the mechanical model analysis and FEM analysis, its simpler coupling beam component, a Y-shaped coupling beam, separates the natural frequency of operation mode from pseudo mode by at least 200 Hz. By optimizing the coupling component between the sense mode motion and the lumped mass twisting motion can be eliminated. Meanwhile, the CSQMG natural frequency distribution is effectively controlled by the optimization flow which is based on a comprehensive derivation of the N-shaped beam, Y-shaped beam, and the effectiveness has been proved by the frequency response curve experiments. The derivation will lay the foundations for the error analysis in further study.

Future works will focus on the circuit optimization, quality factor promotion and MEMS processing improvements. CSQMGs are expected to approach the goal performance of HRGs by increasing the structure thickness. A consistent further study of CSQMG based on rate integrated mode will also be realized.

## Figures and Tables

**Table 1:** Torque expressions of *x_ak_*~*x_fk_*.

	FM1 (a)	FM2 (b)	MM (c)	FB1 (d)	FB2 (e)	MB (f)
**1**	$x_{a1}=\frac{FM1}{2}\left(q+x1\right)$	$x_{b1}=0$	$x_{c1}=-\frac{MM}{2}$	$x_{d1}=0$	$x_{e1}=q+x1$	$x_{f1}=1$
**2**	$x_{a2}=\frac{FM1}{2}\left(q+l1\right)$	$x_{b2}=\frac{FM2}{2}x2$	$x_{c2}=-\frac{MM}{2}$	$x_{d2}=x2$	$x_{e2}=q+l1$	$x_{f2}=1$
**3**	$x_{a3}=\frac{FM1}{2}\left(q+l1+x3\right)$	$x_{b3}=\frac{FM2}{2}l2$	$x_{c3}=-\frac{MM}{2}$	$x_{d3}=l2$	$x_{e3}=q+l1+x3$	$x_{f3}=1$
**4**	$x_{a4}=\frac{FM1}{2}\left(q+l1+l3\right)$	$x_{b4}=\frac{FM2}{2}\left(l2-x4\right)$	$x_{c4}=\frac{-MM}{2}$	$x_{d4}=l2-x4$	$x_{e4}=q+l1+l3$	$x_{f4}=1$
**5**	$x_{a5}=\frac{FM1}{2}\left(q+l1+l3-\frac{x5}{\sqrt{2}}\right)+\frac{FM1}{2}\left(q+l9+l7-\frac{x5}{\sqrt{2}}\right)$	$x_{b5}=0$	$x_{c5}=0$	$x_{d5}=\left(l2-l4-\frac{x5}{\sqrt{2}}\right)+\left(l8-l6-\frac{x5}{\sqrt{2}}\right)$	$x_{e5}=0$	$x_{f5}=2$
**6**	$x_{a6}=-\frac{FM1}{2}\left(q+l9+l7\right)$	$x_{b6}=\frac{FM2}{2}\left(l8-x6\right)$	$x_{c6}=-\frac{MM}{2}$	$x_{d6}=-\left(l8-x6\right)$	$x_{e4}=q+l9+l7$	$x_{f6}=-1$
**7**	$x_{a7}=-\frac{FM1}{2}\left(q+l9+x7\right)$	$x_{b7}=\frac{FM2}{2}l8$	$x_{c7}=-\frac{MM}{2}$	$x_{d7}=-l8$	$x_{e3}=q+l9+x7$	$x_{f7}=-1$
**8**	$x_{a8}=-\frac{FM1}{2}\left(q+l9\right)$	$x_{b8}=\frac{FM2}{2}x8$	$x_{c8}=-\frac{MM}{2}$	$x_{d8}=-x8$	$x_{e2}=q+l9$	$x_{f8}=-1$
**9**	$x_{a9}=-\frac{FM1}{2}\left(q+x9\right)$	$x_{b9}=0$	$x_{c9}=-\frac{MM}{2}$	$x_{d9}=0$	$x_{e1}=q+x9$	$x_{f9}=-1$

**Table 2:** Stiffness error comparison.

**Design Geometric Parameters**	**Beam Number**	**Length/μm**	**Width/μm**
	1	754.5	70
	2	776	34.6
	3	430.5	55
	4	844	43
	5	111	50
**Mode**	**Stiffness/N/m (Calculation)**	**Stiffness/N/m (ANSYS)**	**Percentage Error**
**In-phase Drive**	897.6	904.98	0.82%
**Anti-phase Drive**	834.3	834.03	−0.03%
**Anti-phase Sense**	781.3	866.93	9.88%
**In-Phase Sense**	667.6	703.63	5.12%

**Table 3:** Frequency distribution of each mode.

	Parameter Name	Initial Parameters	After Mode Optimization	After Rotational Coupling Optimization
**Beam Length (mm)**	**L1**	0.68	0.68	0.68
	**L2**	0.755	0.796	0.755
	**L3**	0.4305	0.4305	0.4305
	**L4**	0.844	0.844	0.885
	**L5**	0.1	0.1	0.1
**Beam Width (mm)**	**B1**	0.08	0.072	0.072
	**B2 = B4**	0.04	0.45	0.45
	**B3**	0.05	0.05	0.05
	**B5**	0.024	0.027	0.027
**Mode Frequency (Hz)**	**In-Phase Sen**	5816	6595	6558
	**Pseudo 1**	6205	6932	6923
	**Anti-phase Dri**	6930	7353	7357
	**Anti-phase Sen**	6605	7364	7359
	**Pseudo 2**	7015	7555	7509
	**In-phase Dri**	7071	7645	7570
**Rotational Coupling (1/N)**		0.045	0.022	4.2 × 10^−4^

**Table 4:** Design parameters of the CSQMG.

**Design Parameters**	Structure	Size With Pads	7700 μm × 7700 μm
		Thickness	80 μm
	Oscillator	Single Lumped Mass	0.58 mg
		Beam Width	31 μm~80 μm
		Beam Length	100 μm~825 μm
	Comb Finger	Comb Drive Gap	3.5 μm
		Comb Sense Gap	4 μm~16 μm
		Detection Capacitance	3.3 pF in total (dC/dx = 1.2 μF/m)
	Mechanical Noise Level		0.186°/h/√Hz

**Table 5:** Measured results of the CSQMG.

**Measured Results**	Operating Frequency (After Tuning)	6.8 kHz
	Q factor(20 °C & ≤ 30 Pa)	8500/500 on drive/sense mode
	Scale Factor	20,209.2 LSB/(°/s)
	Scale Factor Nonlinearity	96 ppm
	Measurement Range	±300°/s
	White Noise Level	0.7185°/h/√Hz
	Allan Variance Stability	0.12°/h (@100 s)
